# Family educational pressure, mathematics anxiety, and psychosomatic symptoms: evidence from selected Chinese jurisdictions

**DOI:** 10.3389/fpsyg.2026.1923126

**Published:** 2026-10-12

**Authors:** Ningning Li, Li Gao

**Affiliations:** 1 School of Education, XINYANG College, Xinyang, Henan, China; 2 Student Aid Management Center, Jiangxi Institute of Technology, Nanchang, Jiangxi, China

**Keywords:** adolescent well-being, complex survey design, family educational pressure, Hong Kong, Macao, mathematics anxiety, PISA 2022, psychosomatic symptoms

## Abstract

Adolescent psychological well-being is increasingly recognized as an important dimension of educational quality, particularly in achievement-oriented educational systems where family expectations may be experienced as pressure rather than support. This study examines the relationships among perceived family educational pressure, mathematics anxiety, and psychosomatic symptoms using data from PISA 2022. The initial data extraction covered the complete PISA 2022 public-use student records for comparable Confucian-heritage East Asian systems including Hong Kong (China), Macao (China), and Chinese Taipei (*N* = 16,148). Because psychosomatic-symptom data were unavailable for Chinese Taipei and complete information was required across the focal variables, covariates, and survey weights, the final mediation sample comprised 4,994 students from Hong Kong and Macao. A design-based mediation analysis was conducted using the PISA final student weight and all 80 BRR-Fay replicate weights, controlling for socioeconomic status, gender, grade level, and immigrant background. Family educational pressure was positively associated with mathematics anxiety (B = 0.187, *p* < 0.001), and mathematics anxiety was positively associated with psychosomatic symptoms after adjustment for family pressure and covariates (B = 0.159, *p* < 0.001). Family pressure also retained a positive direct association with psychosomatic symptoms (B = 0.122, *p* < 0.001). The design-based indirect association through mathematics anxiety was statistically distinguishable from zero (ab = 0.0303, 95% CI [0.0217, 0.0388]). The pattern of findings was positive across both participating economies and remained evident after additional adjustment for mathematics achievement using PISA plausible values. Overall, the findings indicate a modest cross-sectional indirect association linking perceived family educational pressure with psychosomatic symptoms through mathematics anxiety. Given the cross-sectional design, the results are interpreted as associations rather than causal effects.

## 1 Introduction

Educational systems are traditionally evaluated through students’ academic achievement, examination performance, progression through schooling, and comparative standing in international large-scale assessments. This policy orientation towards achievement has been bolstered by international tests, like the Program for International Student Assessment (PISA), which have been used to compare the performance of educational systems based on students’ results in mathematics, reading, and science, influencing national policy discussion and reform plans in all the participating systems (Li et al., 2025; OECD, 2023a). Contemporary accounts of educational quality, however, also emphasize students’ psychological well-being, emotional safety, and healthy development (Borgonovi and Pál, 2016; OECD, 2017, 2023b; UNESCO, 2024).

This broader perspective is especially relevant in adolescence. The adolescent years are a period of developmental growth that involve significant changes in the biological, cognitive, emotional and social characteristics of youth, in which they are more likely to be sensitive to being evaluated, compared, to future uncertainty, and to social acceptance (Patton et al., 2016; Sawyer et al., 2018). The World Health Organization estimates that one in seven adolescents (aged 10–19) suffers from a mental disorder (World Health Organization, 2025a, 2025b), and depression, anxiety and behavioral disorders are among the most important causes of illness and disability among this age group. In line with this, Global child-health scholarship also strongly suggests that the family, school, social and policy environments, in which children live and develop, interact and influence adolescent well-being (Clark et al., 2020) and that the school must be recognized as a developmental environment that influences motivation, self-belief, and emotional adjustment, rather than just an educational space (Eccles and Roeser, 2011). Adolescent psychological health is a primary aspect of educational quality because of these concerns.

Academic pressure is one significant route by which educational systems can impact adolescent well-being. Previous studies have shown that academic achievement-related stress, high expectations, and competitive academic settings are linked to anxiety, depressive symptoms, sleep disturbances, and psychosomatic complaints in adolescents (Luthar et al., 2020; OECD, 2017). Research data from the PISA also indicates that students’ wellbeing is strongly linked to their overall school experiences, notions of school self-efficacy and perceived support at school and at home (OECD, 2017; OECD, 2023b). Accordingly, educational pressure is relevant not only to motivation but also to developmental and public-health concerns when it is experienced as persistent or emotionally demanding.

Family educational expectations are especially relevant in this process. Families are one of the most proximal environments that influence adolescents’ academic beliefs, educational aspirations and emotional reactions to success or failure (Bronfenbrenner, 1979; Bronfenbrenner and Morris, 2006). A large and growing body of research demonstrates that parental involvement and educational aspirations are correlated and that parental aspirations are one of the strongest family-level predictors of educational outcomes (Fan and Chen, 2001; Jeynes, 2007; Yamamoto and Holloway, 2010). High expectations can be a signal of confidence and high value for education, and encourage persistence, consistent with expectancy-value accounts of students’ engagement (Wigfield and Eccles, 2000). Therefore, the expectations of the family should not be seen as inherently harmful.

However, family educational expectations and perceived family pressure are conceptually distinct. Expectations refer to parents’ aspirations or desired educational outcomes and may be supportive or motivational. Therefore, literature warns that family involvement is not uniformly beneficial. For example, Pomerantz et al. (2007) suggest that the impacts of parental involvement are related to the actions of involvement, the motivation behind the involvement, and the impact on children’s independence and self-assurance. When adolescents perceive expectations as rigid, conditional, chronic monitoring, and/or fear of disappointing parents, they can be psychologically expensive (Luthar and Becker, 2002; Wang and Sheikh-Khalil, 2014). This difference between supportive expectations and experienced pressure is at the very core in the present study. Family pressure can drive for achievement if it’s presented as encouragement; however, it can turn into a threat to self-worth, family acceptance, or future opportunity if it’s internalized as such.

Mathematics anxiety provides a particularly plausible mechanism through which family pressure may become psychologically consequential. The term Mathematics anxiety has been used to describe the state of being tense, apprehensive, worried, or afraid of mathematics that interferes with learning and performance in mathematics (Ashcraft, 2002; Ashcraft and Ridley, 2005). In many educational contexts mathematics is an academic field with high stakes and a strong symbolic association with academic success, particularly in environments in which mathematics achievement is connected to future academic and occupational opportunities (such as United States) (Sells, 1980). Several systematic reviews and meta-analysis indicate that mathematics anxiety is negatively related to mathematics achievement, confidence, working memory, and tendency towards mathematics related learning path (Barroso et al., 2021; Dowker et al., 2016; Hembree, 1990; Namkung et al., 2019; Suárez-Pellicioni et al., 2016). Importantly, mathematics anxiety is not restricted to low-performing students, but it also emerges among capable, even high-achieving, students when the perceived consequences of failure are high (Foley et al., 2017).

Family pressure is associated with mathematics anxiety when educational outcomes are experienced as carrying high perceived stakes. This interpretation is consistent with cognitive appraisal theory (Lazarus and Folkman, 1984) and with research showing that parents’ mathematical beliefs and anxieties are associated with children’s mathematics anxiety and achievement (Maloney et al., 2015; Ramirez et al., 2018). Adolescent-specific evidence similarly links parent and child mathematics anxiety with adolescents’ mathematics self-efficacy, attitudes, and achievement (Casad et al., 2015). Similarly, PISA-based work also situates mathematics anxiety within wider family, school, and self-belief contexts (OECD, 2013, 2023b). Comparative evidence indicates relatively elevated mathematics anxiety in selected Confucian-heritage systems relative to European comparators (Morony et al., 2013). These studies motivate examination of the proposed association but do not establish a uniform cultural process or a temporal mechanism across the jurisdictions considered here.

Psychosomatic symptoms are an appropriate outcome for examining the psychological costs of academic pressure. Adolescents often express distress through physical complaints such as headaches, stomach pain, fatigue, sleep difficulties, nervousness, and irritability rather than through formal clinical diagnoses (Haugland et al., 2001; Ravens-Sieberer et al., 2008a, 2008b). Such subjective health complaints are widely used in adolescent health research as indicators of stress-related psychological burden and reduced well-being (Haugland et al., 2001; Ravens-Sieberer et al., 2008a, 2008b). Psychosomatic symptoms are especially relevant in educational research because academic stress may become embodied in students’ everyday health experiences even when students continue to perform academically (Ravens-Sieberer et al., 2008a, b). Thus, psychosomatic symptoms provide a meaningful way to capture the hidden emotional and somatic costs that may accompany achievement-oriented schooling.

Despite growing attention to adolescent mental health, several gaps remain. First, high-performing educational systems are still often interpreted primarily through achievement rankings, while the psychological conditions under which achievement is produced receive less attention (Borgonovi and Pál, 2016; OECD, 2017, 2023b). Second, existing research often focuses on school pressure, examination stress, or general academic anxiety, while family pressure remains less clearly distinguished from supportive parental expectations (Pomerantz et al., 2007; Wang and Sheikh-Khalil, 2014). Third, although academic pressure is linked to poorer mental health, fewer studies explain how family-based pressure becomes internalized as a specific academic emotion and then translates into psychosomatic symptoms; a systematic review of academic stress in the final years of secondary school similarly concludes that, while family factors are consistently associated with distress, the specific psychological mechanisms linking family-based pressure to students’ health outcomes remain underspecified (Wuthrich et al., 2020). Fourth, PISA 2022 provides a valuable opportunity to examine adolescent well-being alongside academic and family-context indicators using internationally standardized data, yet this potential remains underused in studies of family pressure and psychological health among Confucian-heritage East Asian populations specifically (OECD, 2023a, 2023b, 2024).

To address these gaps, the present study examines the relationships among perceived family educational pressure, mathematics anxiety, and psychosomatic symptoms using PISA 2022 data. The initial extraction covered three participating economies, while the focal mediation analysis is limited to the economies represented in the final analytic sample. Specifically, the study investigates whether family pressure is associated with psychosomatic symptoms, whether family pressure is associated with mathematics anxiety, whether mathematics anxiety is associated with psychosomatic symptoms, and whether the product of the first two paths constitutes a statistically distinguishable indirect association. Socioeconomic status, gender, grade, and immigrant background are included as covariates, with mathematics achievement added in a plausible-value robustness analysis.

This study makes three contributions. First, it brings student psychological well-being into discussions of educational quality in high-performing East Asian PISA systems represented in the final analytic sample without equating system-level performance with individual academic success. Second, it distinguishes supportive family educational expectations from the student-perceived pressure measured by PISA item ST324Q05JA. Third, it evaluates a theoretically motivated indirect association through mathematics anxiety using PISA design-based estimation, while treating cognitive appraisal theory as the primary interpretive framework and ecological and expectancy-value perspectives as supplementary rather than directly tested theories.

## 2 Literature review and theoretical framework

Educational quality is not merely a measure of outcomes. Nowadays, education policies emphasize psychological well-being too, particularly student well-being, school environment, and social–emotional development (UNESCO, 2024; OECD, 2023b). This issue is specifically pressing during adolescence, the period when a person suffers from increased sensitivity to examination and comparison, uncertainty of the future, and the wish to be accepted by society. According to the data provided by World Health Organization (2025a, 2025b), one out of seven adolescents suffers from mental health disorders, which illustrates the necessity of stimulating environment for families, schools, and communities. These ideas elucidate the need to study thoroughly what the influence of family-related educational requirements on the psychological and somatic state of adolescents is.

Cognitive appraisal theory serves as the main interpretive paradigm for the literature review, with ecological systems theory and expectancy-value theory providing additional perspectives. The former (Lazarus and Folkman, 1984) is particularly pertinent to the postulated relationship, as it focuses on the perception of demands and stress response, but not on the measurement of appraisals and coping skills, which are not included in the current data set. Ecological systems theory (Bronfenbrenner, 1979; Bronfenbrenner and Morris, 2006) situates family communication in the proximal environment of the adolescent, whereas expectancy-value theory (Eccles and Wigfield, 2002) offers contextual terminology for the perceived costs and value of academic outcomes. Several recent studies have found associations between parental academic pressure and perceived costs, burnout, and achievement (e.g., Zhang et al., 2026); however, these constructs are used in the current study as contextual support for the model.

### 2.1 Educational expectations, family pressure, and adolescent health

Educational expectations are not necessarily negative thing. In many cases, they represent care, ambition, investment, and hopes for upward social mobility (Ho, 1996; Li, 2002). Expectancy-value theory (Eccles and Wigfield, 2002) can help understand why expectations might increase the value attributed to academic results, but the cost factor of this theory also takes into account effort, emotional costs, and the costs of missed opportunities. Recent research has shown that perceived academic pressure by parents is positively correlated with several cost factors, and these costs can occur in conjunction with school burnout and achievement (Zhang et al., 2026). However, in the current study, expectancy-value variables are not directly assessed and are considered only as a theoretical background.

The difference between aspiration and pressure is empirically significant. Expectations within the family can be seen as flexible guidance and encouragement; however, these expectations can also be experienced by students as rigid requirements. Analyzing data from China Education Panel Survey, Xu et al. (2024) revealed that high parental expectations were related to lower rates of mental problems if the level of academic pressure was low; at the same time, high expectations and high academic pressure led to poor psychological well-being.

This distinction matters because appraisal is psychological rather than purely structural. The transactional model of stress (Lazarus and Folkman, 1984) suggests that stress is not just the result of demands placed on the person, but of the person’s appraisal of their coping resources in relation to the demands. Primary appraisal involves determining the meaning or importance of the stressor, and secondary appraisal involves assessing coping resources, both of which help to determine if academic pressure results in feelings of distress (Folkman, 2013). The educational expectations can consequently have conflicting effects, depending on how adolescents evaluate them as challenges to tackle, or demands to face that are too difficult for them to handle.

The recent findings from China suggest the contextual link between education-related pressures and psychological distress (Zhou et al., 2025; Hua et al., 2023). For instance, Zhou et al. (2025) found some indirect connections related to parental expectations, stress perception, and core self-worth, whereas Hua et al. (2023) studied the connection between academic pressure, anxiety symptoms, and NR3C1 methylation in Chinese teenagers. It can be used as an insight for the stress literature in general but not as evidence for the current study.

The physiological level of complementary biological evidence supports this pattern. Academic pressure was positively associated with increased anxiety symptoms and with epigenetic changes in NR3C1 gene methylation (the gene that codes for the glucocorticoid receptor, which is the primary molecule in the regulation of stress responses), suggesting that chronic academic pressure may get “under your skin” during the adolescent period and have long-term effects on stress reactivity (Hua et al., 2023).

Existing research in Chinese contexts illustrate how family educational concern can be associated with adolescent adjustment through multiple relational and psychological pathways. Lu F. et al. (2025) found that parental educational anxiety was associated with adolescent problem behaviours through psychological control and parent–child relationship quality. Separate evidence links parental educational anxiety with adolescent depression through moral disengagement and negative coping (Lu Y. et al., 2025). These studies provide contextual evidence about family-related educational stressors, but they are not treated as evidence of prevalence or identical processes in the present analytic settings.

Cultural context is treated cautiously in the present study because the selected PISA jurisdictions and mainland China have distinct institutional and educational settings. Comparative research in Confucian-heritage systems suggests that mathematics anxiety may remain comparatively elevated even in high-performing educational contexts (Morony et al., 2013). Evidence from mainland China is therefore used only as contextual literature rather than as a direct description of the present analytic settings, and the study does not assume a uniform cultural mechanism across these settings.

The motivational and burdensome dimensions of educational expectation have been extensively documented. Using CEPS data, Xu et al. (2024) found that high parental expectations were associated with fewer mental health problems when academic pressure was low, but with more mental health problems when pressure was high, suggesting that the same expectations can produce divergent psychological outcomes depending on how they are communicated. Research on parent–child expectation discrepancies has similarly found that larger discrepancies between parental and adolescent self-expectations are associated with heightened depressive symptoms (Zhang and Jiang, 2024). A longitudinal study of German secondary school students found that when parents’ aspirations exceeded their own achievement expectations—what the authors term “over-aspiration”—this pattern predicted greater anxiety and lower levels of pride in the same year, highlighting complex temporal relations between parental expectations and students’ achievement emotions (Sakaki et al., 2025).

Taken together, the theoretical and empirical evidence reviewed above supports the expectation that family pressure should show a positive association with adverse psychological health outcomes among adolescents. Although educational expectations can serve as motivational resources when experienced as support, pressure may be associated with psychological distress in high-stakes educational contexts. This motivates the first hypothesis:

*H1*: Family pressure is positively associated with psychosomatic symptoms.

### 2.2 Mathematics anxiety as a potential indirect pathway

Academic anxiety is one of the most likely scenarios in which academic pressure can have psychological ramifications. Failure is not the only source for anxiety; it can also manifest itself in anticipation of evaluation, comparison and perceived lack of success, even among otherwise successful students (Zeidner, 1998). Mathematics anxiety has been defined as a tension, apprehension or fear which disrupts the handling of numbers and the solution of mathematical problems in a variety of academic and life situations (Ashcraft and Ridley, 2005), and is known to have quantifiable personal, educational and cognitive consequences (Ashcraft, 2002). Math anxiety consists of cognitive components (worries, rumination and negative self-talk) and affective (tension and physiological arousal) components that negatively affect mathematical performance and learning (Carey et al., 2016; Dowker et al., 2016). A study of senior secondary students found that family pressure, inappropriate teaching methods, the school environment, neurological responses to stress, and developmental stage are significantly related to mathematics anxiety, while learners’ behavior, genetic predispositions, and gender were not significant predictors in that context (Asanre et al., 2025).

Preliminary evidence from meta-analyses shows that the relationship between mathematics anxiety and mathematics performance is bidirectional and that the evaluative threat of competitive educational contexts may be especially strong (Barroso et al., 2021; Namkung et al., 2019). Importantly, mathematics anxiety does not only affect students who are less able to do math; capable students could also be more anxious because of the high stakes of failure in math (Barroso et al., 2021). The combined presence of academic competence and anxiety highlights the need to explore the domain-specific emotional responses as possible mediators of other psychosocial outcomes. Aaviste et al. (2026) used the data from PISA 2022 to determine that stress resistance (*β* = −0.17), perseverance (*β* = −0.15) and emotional control (*β* = −0.12) were significant predictors of lower levels of mathematics anxiety, whereas perceived lesson quality (*β* = −0.29) and teacher support (*β* = −0.07) were significant protective factors.

At the theoretical level, mathematics anxiety is compatible with the proposed indirect pathway. Cognitive appraisal theory (Lazarus and Folkman, 1984) suggests that anxiety can arise when situations are evaluated as consequential or threatening relative to perceived coping resources. Applied to mathematics, students may experience greater anxiety when family-linked expectations heighten the perceived stakes of performance. Contextual evidence from Asanre et al. (2025) likewise indicates that family pressure, teaching practices, and school environment are associated with mathematics anxiety. These findings motivate the proposed association, but they do not demonstrate the appraisal process itself because appraisal and coping resources are not measured in the present data.

Expectancy-value theory provides supplementary context for why educational expectations may matter emotionally. Greater perceived importance and cost can be associated with apprehension when students doubt their ability to meet valued standards (Eccles and Wigfield, 2002). Recent work by Zhang et al. (2026) similarly links perceived parental academic pressure with multiple cost dimensions and school burnout. These constructs are not measured in the present PISA model, so they are used only to contextualize why family pressure may covary with mathematics anxiety rather than as evidence of a tested causal process.

Previous school-based anxiety studies suggest that evaluative messages, threat messages, and fear of failure are factors that affect emotional functioning. The previously mentioned relationships between reduced perceived control over academic outcomes and anxious responding were identified as systematic in Putwain’s (2019a, 2019b) structural model of test anxiety, revealing that anxious responses are part of a complex network of motivational and self-protective processes that include self-handicapping, which involved self-imposed barriers to success to ensure an external explanation for failure. Cross-cultural evidence indicated that a growth mindset generally led to decreased mathematics anxiety in all four systems, with the mediating pathways varying by country: competence was the most consistent universal mediator, and relatedness mediated stronger in collectivist countries (South Korea and Singapore), as would be particular in Confucian-heritage varieties of education, like the ones studied in the present research study.

Evidence from comparable Confucian-heritage East Asian systems is more relevant to the present contextual argument. In a cross-national comparison, Morony et al. (2013) reported relatively elevated mathematics anxiety in several such systems compared with European systems. Such findings motivate examination of family educational pressure in the present context, but they do not establish that peer-comparison norms, family processes, or the meaning of mathematics operate identically across economies.

Recent research further supports a family–anxiety association. In a survey of secondary students in Nigeria, Asanre et al. (2025) reported significant associations between mathematics anxiety and contextual factors including family pressure, teaching practices, and the school environment. Although that context differs from the East Asian PISA settings considered here, the findings are consistent with treating family pressure as one possible correlate of mathematics anxiety. This interpretation is also compatible with evidence that parental psychological control (i.e., parenting practices that intrude upon the child’s psychological autonomy) is associated with adolescent internalising symptoms across multiple cultural contexts (Barber et al., 2005; Soenens and Vansteenkiste, 2010). Using PISA 2022 data from Estonia, Aaviste et al. (2026) found that greater emotional control and stress resistance were associated with lower mathematics anxiety, providing contextual evidence that social–emotional resources covary with mathematics anxiety.

The internalization of parents’ expectations through contingent self-worth could have significant psychological implications. Once academic success becomes an indicator of acceptance or worthiness of one’s person, academic failures may come to threaten self-worth (Crocker and Wolfe, 2001). The students whose self-worth is highly contingent upon academic success display greater self-esteem fluctuation and negative affect after academic failure (Crocker et al., 2003). This phenomenon is particularly applicable to mathematics since math skills serve as the primary indicator of competence in the subject area (Ashcraft and Ridley, 2005). Similarly, Lu Y. et al. (2025) discovered that parents’ educational anxiety correlated with adolescent depression through moral disengagement and negative coping strategies, however, their cross-sectional design does not allow for causal interpretation.

The choice of mathematics anxiety as the focal mediator is theoretically motivated but is not assumed to be unique. The family-pressure item is domain-general and does not itself refer to mathematics. ANXMAT was privileged because mathematics was the major assessment domain in PISA 2022, the cycle provides a well-documented domain-specific mathematics-anxiety index, and Hong Kong and Macao are among the high-performing systems in which mathematics anxiety remains substantively salient (OECD, 2023a; OECD, 2024a). Accordingly, the mediator choice reflects both theory and the measurement opportunities of PISA 2022. The present model does not establish that mathematics anxiety is the only, or necessarily the strongest, emotional pathway; future work should compare it with general academic, test, or other domain-specific anxiety measures where comparable data are available.

Based on this literature, the study tests whether family pressure is positively associated with mathematics anxiety (H2) and whether mathematics anxiety is positively associated with psychosomatic symptoms (H3). These hypotheses concern cross-sectional associations and do not assume temporal causation:

*H2*: Family pressure is positively associated with mathematics anxiety.

*H3*: Mathematics anxiety is positively associated with psychosomatic symptoms.

### 2.3 Psychosomatic symptoms as an Indicator of psychological health

Adolescent psychological distress frequently manifests in embodied form. When dealing with emotional stress, headaches, stomach pain, fatigue, sleep disturbances, and other physical symptoms are often experienced (Haugland et al., 2001). These are particularly relevant to adolescent research as they reflect distress that may not be clinically or diagnostically described, but that represents reduced psychological health nevertheless. The significant relationship between psychosomatic symptoms and school-related stress, school pressure and perceived lack of social support shown by Haugland et al. (2001) illustrates that subjective health complaints are not a trivial background noise in adolescence, but have meaning as a measure of stress related burden.

Academic pressure is consistently associated with psychosomatic complaints. Using a national Swedish sample of 3,745 participants aged 11 to 15 years from the Health Behavior in School-Aged Children (HBSC) survey, Birgersson et al. (2024) identified a positive graded association between school pressure and psychosomatic complaints. Physical activity was associated with fewer complaints overall, but it did not significantly moderate the school-pressure association. In keeping with this broader pattern, Ravens-Sieberer et al. (2008a, 2008b) documented the cross-national prevalence and dimensionality of subjective health complaints among adolescents. At the population level, adolescent mental-health problems are also a major public-health concern; World Health Organization (2025a, 2025b) estimates that approximately one in seven 10- to 19-year-olds experiences a mental disorder.

Psychosomatic complaints are developmentally relevant because adolescents may express distress through physical as well as emotional symptoms (Haugland et al., 2001; Rask et al., 2016). Cultural norms surrounding emotional expression can also shape how distress is reported. For the present study, psychosomatic symptoms are therefore treated as a non-clinical indicator of subjective health burden rather than as a psychiatric diagnosis. Longitudinal evidence indicates that somatic symptoms reported during adolescence can have prognostic relevance for later mental health. In a community-based follow-up study, Bohman et al. (2018) found that adolescent somatic symptoms predicted later hospital-based care for severe adult mental illness, including mood disorders, even after adjustment for important adolescent mental-health factors. This literature supports treating recurrent psychosomatic symptoms as clinically relevant indicators of adolescent burden, while the present cross-sectional PISA analysis does not itself establish long-term consequences.

Psychosomatic symptoms are relevant to educational research because school pressure has been associated with greater psychosomatic complaints in adolescent populations (Birgersson et al., 2024). Such complaints can coexist with continued academic participation and therefore capture aspects of student well-being that achievement indicators do not. This does not imply that high system-level performance itself causes poorer health; rather, it motivates examination of specific pressures experienced by students. The present study treats psychosomatic symptoms as an indicator of psychological and somatic burden that is developmentally appropriate for adolescents. The outcome is operationalized using the PISA PSYCHSYM index, which draws on a symptom checklist closely related to measures used in international adolescent health research (Haugland et al., 2001; OECD, 2024a). This provides a standardized non-clinical outcome suitable for examining associations between educational pressure and students’ subjective health complaints.

### 2.4 Integrated theoretical framework

Cognitive appraisal theory (Lazarus and Folkman, 1984) is the primary framework for interpreting the proposed statistical pathway. It suggests that psychological responses depend partly on how demands are evaluated relative to coping resources. Ecological systems theory (Bronfenbrenner, 1979; Bronfenbrenner and Morris, 2006) is used to locate family communication within the adolescent’s proximal environment, while expectancy-value theory (Eccles and Wigfield, 2002) provides supplementary context for why academic outcomes may carry high subjective importance and cost. Because appraisal, control, task value, and perceived cost are not directly measured, the analyses test associations consistent with these perspectives rather than the theories themselves.

This integrated framework yields four testable hypotheses, summarized in Table 1 and examined empirically in the sections that follow.

**Table 1:** Hypothesized Relationships Derived from the Integrated Theoretical Framework.

Hypothesis	Statement	Theoretical Basis
H1	Family pressure is positively associated with psychosomatic symptoms.	Ecological systems theory (Bronfenbrenner, 1979); cognitive appraisal theory (Lazarus and Folkman, 1984); empirical evidence (Zhou et al., 2025; Xu et al., 2024; Lu F. et al., 2025; Wang et al., 2025)
H2	Family pressure is positively associated with mathematics anxiety.	Expectancy-value theory (Eccles and Wigfield, 2002); cognitive appraisal theory (Lazarus and Folkman, 1984); empirical evidence (Asanre et al., 2025; Zhang et al., 2026; Aaviste et al., 2026)
H3	Mathematics anxiety is positively associated with psychosomatic symptoms.	Cognitive appraisal theory (Lazarus and Folkman, 1984); subjective health literature (Haugland et al., 2001); empirical evidence (Birgersson et al., 2024; Bohman et al., 2018)
H4	Mathematics anxiety is statistically associated with an indirect pathway between family pressure and psychosomatic symptoms.	Integrated framework (H1–H3)

Developed from Bronfenbrenner (1979), Eccles and Wigfield (2002), Lazarus and Folkman (1984), and supporting empirical evidence.

#### 2.4.1 Conceptual pathway

The theoretical model can be summarized as a proposed statistical pathway. Ecological systems theory (Bronfenbrenner, 1979) locates family communication within the adolescent’s proximal environment; cognitive appraisal theory (Lazarus and Folkman, 1984) provides the primary interpretation for why perceived demands may be associated with anxiety; and expectancy-value theory (Eccles and Wigfield, 2002) provides supplementary context for the perceived importance and cost of academic outcomes. Empirically, the model tests whether family pressure is associated with mathematics anxiety, whether mathematics anxiety is associated with psychosomatic symptoms, and whether their product forms a statistically distinguishable indirect association. The direct family-pressure association with psychosomatic symptoms is also estimated. Because appraisal, coping, task value, and cost are not directly measured, these theories guide interpretation rather than constituting measured mechanisms (Table 2).

**Table 2:** PISA 2022 student records and common mediation sample by economy.

Economy	Public-use records extracted	Complete mediation cases	Analytic role
Hong Kong (China)	5,907	2,749	Included in mediation analysis
Macao (China)	4,384	2,245	Included in mediation analysis
Chinese Taipei	5,857	0	PSYCHSYM unavailable in analytic file
Total	16,148	4,994	All selected-economy records retained at extraction

All public-use student records available for the three selected economies were retained at extraction. The design-based mediation analysis uses all complete observations on the required model variables and weights: Hong Kong (China) = 2,749 and Macao (China) = 2,245 (*n* = 4,994). Chinese Taipei contributes no cases to the mediation model because PSYCHSYM was unavailable in the analytic file.

Recent empirical studies provide patterns that are broadly consistent with elements of the integrated interpretation. Zhou et al. (2025) linked parental expectations, perceived stress, core self-worth, and psychological distress among Chinese youth; Lu Y. et al. (2025) identified indirect associations involving parental educational anxiety, moral disengagement, negative coping, and adolescent depression; and Putwain (2019a, 2019b) examined appraisal-related processes in test anxiety. Taken together, these studies are compatible with the present framework, but they do not directly test all of its component constructs in the current PISA sample.

## 3 Methodology

This section describes the research design, data source, sampling procedure, operationalization of variables, analytical model, and estimation strategy employed in the present study. Each decision is reported with reference to the methodological literature and to the specific characteristics of the PISA 2022 database to enable replication and independent evaluation.

### 3.1 Research design

This study adopts a quantitative, cross-sectional explanatory design using secondary PISA 2022 survey data. The design is quantitative because the focal constructs are operationalized through numerically coded questionnaire items and OECD-constructed indices, and cross-sectional because all focal measures were observed within the same assessment cycle. “Explanatory” is used in the limited statistical sense that the study evaluates a pre-specified indirect-association model; the design does not establish temporal order or causal mediation.

A mediation decomposition was selected to quantify whether the association between family pressure and psychosomatic symptoms contains a statistically distinguishable component associated with mathematics anxiety. In cross-sectional data, this decomposition is descriptive of an indirect statistical pathway and should not be interpreted as proof that mathematics anxiety temporally intervenes between family pressure and psychosomatic symptoms (Hayes, 2022).

### 3.2 Data source

Data were drawn from the Program for International Student Assessment (PISA) 2022 public-use database. PISA is a triennial large-scale international assessment of 15-year-old students in mathematics, reading, and science, accompanied by questionnaires on student experiences and learning environments (OECD, 2023a). The selected student files and supporting codebooks are publicly available through the OECD PISA 2022 database repository (OECD, 2024b), while sampling, scaling, weighting, and variance-estimation procedures are documented in the PISA 2022 Technical Report (OECD, 2024a).

The PISA 2022 cycle is particularly appropriate for the present study because the student questionnaire contains the focal family-pressure, mathematics-anxiety, and psychosomatic-symptom measures used here (OECD, 2024a, 2024b). In addition, PISA 2022 Volume II gives substantial attention to learning environments, support from home, and student well-being (OECD, 2023b). These features permit analysis of family educational pressure and psychological health within a large international survey while retaining the survey’s design information.

### 3.3 Target population and economy selection

The initial data extraction covered the complete PISA 2022 public-use student files for Hong Kong (China), Macao (China), and Chinese Taipei (*N* = 16,148). The economies were selected because each participated independently in PISA 2022. For the focal mediation analysis, however, psychosomatic-symptom data were unavailable for Chinese Taipei in the analytic file. Consequently, the common complete-case sample comprises Hong Kong and Macao only. The inferential scope of the mediation findings is therefore limited to these two economies.

### 3.4 Sampling procedure

PISA 2022 uses a stratified two-stage probability sampling design. Schools are sampled within explicit strata, with school selection probabilities linked to size, and eligible 15-year-old students are then sampled within participating schools. Because selection probabilities and subsequent weighting adjustments vary, design-based analysis requires the PISA final student weight together with replicate weights to obtain population-relevant estimates and sampling variances (OECD, 2024a). The present analysis therefore does not treat the 4,994 complete cases as a simple random sample.

The present study retained all 16,148 records available in the three selected public-use files at the extraction stage consisting of: Hong Kong (China) = 5,907, Macao (China) = 4,384, and Chinese Taipei = 5,857. The final mediation sample contains every observation with complete data on the focal variables, covariates, and final student weight (*n* = 4,994: Hong Kong = 2,749; Macao = 2,245). Thus, case reduction in the mediation analysis reflects variable availability and complete-case requirements rather than a researcher-imposed sampling fraction.

### 3.5 Measures and operationalization

All focal constructs and covariates were derived from existing PISA 2022 student questionnaire items and OECD-constructed index variables. The conceptual model is: Family Pressure (X) → Mathematics Anxiety (M) → Psychosomatic Symptoms (Y), with socioeconomic status, gender, grade, and immigrant status as covariates. Table 3 summarizes each variable’s role, PISA code, operationalization, and item source.

**Table 3:** Operationalization of focal variables and covariates.

Role in model	Construct	PISA variable	Operationalization and scale	Items/Source
Independent variable (X)	Family pressure (FPRESS)	ST324Q05JA	Single student-report item: agreement with ‘I feel pressure from my family to follow a specific path (e.g., go to college, work in the family business, learn a trade) after [the final year of compulsory education].’ Four-category agreement scale (Strongly disagree to Strongly agree).	1 item; PISA 2022 Student Questionnaire/public-use documentation (OECD, 2024a, 2024b)
Mediator (M)	Mathematics anxiety (ANXMAT)	ANXMAT	OECD-constructed IRT/WLE-scaled ANXMAT index from six ST292 mathematics-anxiety items. Public-use item codes: 1 = Strongly agree, 2 = Agree, 3 = Disagree, 4 = Strongly disagree; the released scale is oriented so that higher ANXMAT scores indicate greater mathematics anxiety.	6 items; PISA 2022 index documentation (OECD, 2023a, 2023b; OECD, 2024a, 2024b)
Dependent variable (Y)	Psychosomatic symptoms (PSYCHSYM)	PSYCHSYM	OECD-constructed WLE index from nine WB154 psychosomatic-symptom items. Five frequency categories: Rarely or never, About every month, About every week, More than once a week, About every day; higher scores = greater symptom burden.	9 items; PISA 2022 Well-Being Questionnaire/Technical Report (OECD, 2024a, 2024b)
Covariate	Socioeconomic Status (SES/ESCS)	ESCS	OECD composite index of family wealth, home educational resources, and parental occupation/education; standardized; higher = higher SES	Composite; PISA 2022 (OECD, 2024a, 2024b)
Covariate	Gender (GEND)	ST004D01T	Binary item: 1 = female, 2 = male (as coded in PISA database)	1 item; PISA 2022 (OECD, 2024a, 2024b)
Covariate	Grade (GRD)	GRADE	PISA relative-grade index: 0 = the country/economy modal grade for 15-year-olds; negative values indicate grades below the modal grade and positive values indicate grades above it. Included to control for within-economy grade-position variation.	Derived; PISA 2022 Technical Report (OECD, 2024a)
Covariate	Immigrant Status (IMM)	Derived immigrant variable	PISA-derived categorical variable indicating first-generation, second-generation, or native-born status; included to control for documented associations between immigrant background and both academic anxiety and health outcomes	Derived; PISA 2022 (OECD, 2024a, 2024b)

HBSC = health behavior in school-aged children symptom checklist. IRT = item response theory. ANXMAT and PSYCHSYM are OECD-constructed indices. Questionnaire and index documentation is provided in the PISA 2022 Technical Report and public-use database/codebooks (OECD, 2024a, 2024b).

*Family pressure (FPRESS)*: The independent variable is PISA item ST324Q05JA: “I feel pressure from my family to follow a specific path (e.g., go to college, work in the family business, learn a trade) after [the final year of compulsory education].” Responses are recorded on a four-category agreement scale. The item therefore measures the student’s perceived pressure to follow a family-linked post-compulsory-education path; it is not treated as a general measure of parental aspirations or supportive expectations.

Mathematics anxiety (ANXMAT): The mediator is the OECD-constructed ANXMAT index derived from six ST292 items concerning mathematics anxiety. The items ask about worry that mathematics classes will be difficult, tension when doing mathematics homework, nervousness when doing mathematics problems, helplessness when doing a mathematics problem, worry about poor mathematics [marks], and anxiety about failing mathematics. In the public-use file, the item response codes are 1 = Strongly agree, 2 = Agree, 3 = Disagree, and 4 = Strongly disagree; the released ANXMAT WLE scale is oriented so that higher scores indicate greater mathematics anxiety (OECD, 2023a; OECD, 2024a). Complete item wording is reproduced in Appendix A.

Psychosomatic symptoms (PSYCHSYM): The dependent variable is the OECD-constructed PSYCHSYM WLE index based on nine WB154 items asking how often, during the past 6 months, students experienced headache, stomach pain, back pain, feeling depressed, irritability or bad temper, feeling nervous, difficulties in getting to sleep, feeling dizzy, and feeling anxious. Responses use five frequency categories (Rarely or never, About every month, About every week, More than once a week, About every day). Higher scores indicate more frequent psychosomatic complaints (OECD, 2024a, 2024b). Complete item wording and the response scale are reproduced in Appendix A.

Covariates: Socioeconomic status was represented by the OECD ESCS index. Gender was coded using ST004D01T (1 = female, 2 = male). Grade was represented by the PISA GRADE relative-grade index: 0 denotes the modal grade for 15-year-olds in the country/economy, negative values indicate grades below the modal grade, and positive values indicate grades above it (OECD, 2024a). Immigrant background was treated as nominal and dummy-coded with native students as the reference category (second-generation and first-generation indicators). Mathematics achievement was examined in a robustness analysis using all ten PISA mathematics plausible values and the same complex-survey weighting procedure.

#### 3.5.1 Measurement quality and cross-economy comparability

The three focal variables differ in measurement form. FPRESS is a single observed item and therefore has no internal-consistency coefficient, whereas ANXMAT and PSYCHSYM are OECD-calibrated multi-item IRT/WLE indices. Sample-specific internal consistency was evaluated directly in the 4,994-case analytic file using the final student weight (W_FSTUWT). For ANXMAT, the item-level data show a structured missing pattern: 4,978 of 4,994 students answered five of the six ST292 items and no student answered all six. A conventional listwise alpha is therefore undefined; as a transparent descriptive check, Cronbach’s alpha was calculated from the design-weighted pairwise-complete covariance matrix. The resulting alpha was 0.922 for the combined analytic sample, 0.925 for Hong Kong, and 0.896 for Macao. For PSYCHSYM, design-weighted complete-case alpha was 0.903 in the combined sample (*n* = 4,864), 0.904 in Hong Kong (*n* = 2,684), and 0.890 in Macao (*n* = 2,180). These values indicate good internal consistency in the analytic context. A study-specific multigroup CFA or formal measurement-invariance test across Hong Kong and Macao was not conducted. Consequently, coefficient magnitudes across FPRESS, ANXMAT, and PSYCHSYM should not be interpreted as directly comparable indicators of construct strength. Moreover, the equal-economy Table Average is treated as a descriptive summary rather than evidence of measurement equivalence.

### 3.6 Analytical model and estimation strategy

The primary analysis used design-based linear regression and mediation decomposition appropriate for PISA data. Models were estimated with the PISA final student weight (W_FSTUWT) and all 80 student replicate weights (W_FSTURWT1-W_FSTURWT80), using PISA balanced repeated replication with Fay adjustment. The mediator model regressed mathematics anxiety on family pressure and covariates; the outcome model regressed psychosomatic symptoms on family pressure, mathematics anxiety, and covariates; and a third model estimated the total association without the mediator. Models were estimated separately for the two economies represented in the final analytic sample. The IEA IDB Analyzer “Table Average” is an equal-economy summary and is reported as such; it is not interpreted as a population-pooled coefficient.

The mediation model comprises two simultaneous regression equations. The first equation (the mediator model) regresses mathematics anxiety on family pressure and all covariates:

$$\begin{array}{l} M_{i}=\alpha_{0}+aX_{i}+\gamma ₁{\text{ESCS}}_{i}+\gamma ₂{\text{GENDER}}_{i}+\gamma ₃{\text{GRADE}}_{i}\\ +\gamma ₄\text{IMMIG}\_{\text{SECOND}}_{i}+\gamma_{5}\text{IMMIG}\_{\text{FIRST}}_{i}+\varepsilon_{i} \end{array}$$

Where *a* is the path-a coefficient representing the association between family pressure and mathematics anxiety. The second equation (the outcome model) regresses psychosomatic symptoms on both family pressure and mathematics anxiety, plus all covariates:

$$\begin{array}{l} Y_{i}=\beta_{0}+c^{\prime }X_{i}+bM_{i}+\delta ₁{\text{ESCS}}_{i}+\delta ₂{\text{GENDER}}_{i}+\delta ₃{\text{GRADE}}_{i}\\ +\delta ₄\text{IMMIG}\_{\text{SECOND}}_{i}+\delta_{5}\text{IMMIG}\_{\text{FIRST}}_{i}+\varepsilon_{i} \end{array}$$

Where b is the path-b coefficient representing the association between mathematics anxiety and psychosomatic symptoms, controlling for family pressure, and c′ is the direct effect of family pressure on psychosomatic symptoms, controlling for the mediator. A third equation estimates the total effect model by regressing psychosomatic symptoms on family pressure and covariates without the mediator:

$$\begin{array}{l} Y_{i}=\tau_{0}+cX_{i}+\pi ₁{\text{ESCS}}_{i}+\pi ₂{\text{GENDER}}_{i}+\pi ₃{\text{GRADE}}_{i}\\ +\pi ₄\text{IMMIG}\_{\text{SECOND}}_{i}+\pi_{5}\text{IMMIG}\_{\text{FIRST}}_{i}+\varepsilon_{i} \end{array}$$

The indirect association was calculated within each economy as the product of the economy-specific path-a and path-b coefficients and then summarized as the equal-economy average, consistent with the IDB Analyzer reporting framework. Thus, the reported Table-Average indirect association is the average of the two economy-specific products, not the product of the displayed Table-Average path coefficients. This distinction matters because the product of averages need not equal the average of products. To preserve the PISA survey design, the economy-specific product term was recomputed under each of the 80 replicate weights, and the equal-economy sampling variance was derived from the BRR-Fay replicate distribution. A 95% confidence interval excluding zero was taken as evidence of a statistically distinguishable indirect association. The proportion mediated (ab/c) is reported descriptively and is not interpreted causally.

For the mathematics-achievement robustness analysis, the same model was re-estimated using all ten PISA mathematics plausible values as an additional covariate, with estimates combined across plausible values while retaining the final and replicate weights. This avoids treating a single plausible value as an error-free observed achievement score.

Because the study is cross-sectional, mediation terminology refers to statistical decomposition of associations and not to an established temporal or causal mechanism. The inferential emphasis is therefore on the indirect association and its design-based confidence interval, rather than on a mechanical “partial” versus “full” mediation classification.

Primary model coefficients are reported as unstandardized B estimates, with statistical significance evaluated at alpha = 0.05. Economy-specific estimates are presented alongside the equal-economy summary. For effect-size interpretation, fully standardized focal coefficients were derived from the economy-specific design-weighted standard deviations and the corresponding design-based B estimates. These standardized coefficients are reported descriptively in the Results. Conventional OLS diagnostics were also examined on the common analytic sample as supplementary checks for multicollinearity, residual behavior, leverage, and influential observations.

### 3.7 Missing data and effective sample sizes

Our initial extraction contained 16,148 student records. The availability of variable varied across constructs (i.e., family pressure *n* = 8,554; mathematics anxiety *n* = 15,582; psychosomatic symptoms *n* = 9,808; ESCS *n* = 15,765; grade *n* = 16,148). The common design-based mediation sample consists of 4,994 complete records. This reduction in the sample reflects a combination of PISA questionnaire availability/routing and item-level missingness rather than a subsampling fraction by researcher. No data imputation was used, and all observations that were complete on the focal variables, covariates, and final student weight were retained in the sample.

The common sample consisted of 2,749 students from Hong Kong and 2,245 from Macao. Chinese Taipei did not contribute observations to the mediation analysis because PSYCHSYM was not available in the analytic file. To evaluate whether complete-case selection among the two analyzable economies was associated with observed student characteristics, the pre-complete-case file was matched to the final 4,994-case analytic file using the unique student identifier. Among the 10,291 Hong Kong/Macao records, 4,994 (48.5%) entered the mediation sample. The remaining 5,297 observations were excluded from the sample because one or more required variables were incomplete. Design-weighted comparisons using W_FSTUWT showed only small observed differences: mean ESCS was −0.443 in included cases versus −0.478 in excluded cases (standardized mean difference [SMD] = 0.036); the weighted proportion female was 48.4% versus 45.7% (SMD = 0.054); and mean relative grade was −0.406 versus −0.422 (SMD = 0.025). Economy-specific comparisons were also small (all absolute SMDs ≤ 0.062). These diagnostics reduce concern about marked selection on the observed covariates examined. However, they do not establish that data are missing completely at random or rule out differences on unobserved characteristics. Table 4 summarizes the comparison.

**Table 4:** Design-weighted comparison of included and excluded cases in Hong Kong and Macao.

Characteristic	Included (*n* = 4,994)	Excluded (*n* = 5,297)	SMD
ESCS, weighted M	−0.443	−0.478	0.036
Female, weighted %	48.4	45.7	0.054
Relative grade, weighted M	−0.406	−0.422	0.025

The comparison is restricted to Hong Kong and Macao because PSYCHSYM was structurally unavailable for Chinese Taipei. Estimates use the PISA final student weight (W_FSTUWT). SMD = standardized mean difference, computed as included minus excluded. The initial extraction contained 16,148 records; 5,857 Chinese Taipei records were structurally ineligible for the focal mediation model, leaving 10,291 Hong Kong/Macao records for the complete-case attrition comparison. Economy-specific absolute SMDs ranged from 0.021 to 0.062.

### 3.8 Ethical considerations

This study is based entirely on secondary analysis of publicly available PISA 2022 data released by the OECD for research use. The original PISA 2022 data were collected under OECD/PISA procedures and ethical protocols; the present study involved only secondary analysis of anonymized public-use data. The public-use files contain no direct personal identifiers, and the present authors conducted no primary data collection and had no contact with participants. The manuscript reports the study’s institutional review status as not applicable; researchers should nevertheless follow any local institutional requirements governing secondary analysis of public-use data (OECD, 2024a, 2024b).

## 4 Results

### 4.1 Descriptive statistics

Weighted descriptive statistics for the common analytic sample were: family pressure M = 2.329 (SD = 0.830), mathematics anxiety M = 0.238 (SD = 1.125), psychosomatic symptoms M = −0.204 (SD = 0.994), ESCS M = −0.450 (SD = 0.960), and grade M = −0.391 (SD = 0.628). Table 5 reports zero-order Pearson correlations among the focal variables and covariates. The focal correlations were positive but modest: family pressure with mathematics anxiety, *r* = 0.113; family pressure with psychosomatic symptoms, *r* = 0.118; and mathematics anxiety with psychosomatic symptoms, *r* = 0.229. In practical terms, the focal zero-order correlations are small to modest (*r* = 0.113–0.229), and the primary mediator and outcome models explain modest proportions of variance; the statistical significance of the design-based estimates should therefore not be read as evidence of large effects.

**Table 5:** Zero-order correlations among focal variables and covariates.

Variable	Family pressure	Mathematics anxiety	Psychosomatic symptoms	ESCS	Grade
1. Family pressure	1				
2. Mathematics anxiety	0.113***	1			
3. Psychosomatic symptoms	0.118***	0.229***	1		
4. ESCS	0.001	−0.058***	0.070***	1	
5. Grade	−0.017	0.019*	0.070***	0.119***	1

Correlations are conventional zero-order Pearson estimates reported descriptively; pairwise N varies because of variable availability. Statistical inference for the focal hypotheses is based on the PISA final student weight and 80 BRR-Fay replicate weights. *p < 0.05, ***p < 0.001.

Supplementary regression diagnostics indicated no material multicollinearity or influential-case problem. In the mediator model, the maximum VIF was 1.224 (minimum tolerance = 0.817), maximum Cook’s distance was 0.0059, and maximum leverage was 0.0061. In the outcome model, the maximum VIF was 1.233 (minimum tolerance = 0.811), maximum Cook’s distance was 0.0084, and maximum leverage was 0.0075. Residual plots showed modest departures from normality, but no pattern indicating that the substantive conclusions were driven by a small number of observations.

### 4.2 Mediation model estimates

Design-based mediation models were estimated on 4,994 complete observations using PISA final and replicate weights. Results are reported separately for the two economies represented in the final analytic sample and as the IEA IDB Analyzer equal-economy Table Average. The equal-economy summary is used for the main hypothesis statements, while economy-specific estimates are retained to show cross-economy consistency (Tables 6–8).

**Table 6:** Design-based regression estimates for the mediation model.

Model/Economy	Predictor	B	SE	*t*	*p*	*R* ^2^	Interpretation
Mediator: table average	FPRESS (a)	0.187	0.021	9.103	<0.001	0.072	Positive
Mediator: Hong Kong	FPRESS	0.152	0.030	4.987	<0.001	0.063	Positive
Mediator: Macao	FPRESS	0.222	0.028	8.052	<0.001	0.081	Positive
Outcome: table average	FPRESS (c′)	0.122	0.016	7.693	<0.001	0.102	Positive
Outcome: table average	ANXMAT (b)	0.159	0.014	11.105	<0.001	0.102	Positive
Outcome: Hong Kong	FPRESS (c′)	0.142	0.019	7.388	<0.001	0.092	Positive
Outcome: Hong Kong	ANXMAT	0.147	0.020	7.271	<0.001	0.092	Positive
Outcome: Macao	FPRESS (c′)	0.103	0.025	4.065	<0.001	0.112	Positive
Outcome: Macao	ANXMAT	0.172	0.020	8.430	<0.001	0.112	Positive
Total: table average	FPRESS (c)	0.153	0.017	—	<0.001	—	Positive

*N* = 4,994 complete cases (Hong Kong = 2,749; Macao = 2,245). Coefficients are unstandardized. Estimates and standard errors use W_FSTUWT and 80 BRR-Fay replicate weights. Native students are the reference group for immigrant-background indicators. “Table Average” denotes the equal-economy average reported by IDB Analyzer and should not be interpreted as a population-pooled coefficient.

**Table 7:** Summary of direct, total, and indirect associations.

Association	Estimate	SE	*t*	*p*	95% CI LL	95% CI UL
Path a: FPRESS → ANXMAT	0.187	0.021	9.103	<0.001	—	—
Path b: ANXMAT → PSYCHSYM	0.159	0.014	11.105	<0.001	—	—
Direct association (c′)	0.122	0.016	7.693	<0.001	—	—
Total association (c)	0.153	0.017	—	<0.001	—	—
Indirect association (ab)	0.0303	0.0044	—	—	0.0217	0.0388
Proportion mediated	0.198	—	—	—	—	—

CI = design-based 95% confidence interval. The indirect-effect standard error and confidence interval were derived from the 80 BRR-Fay replicate-weight estimates. Not statistic or conventional p value is reported for the indirect association because inference is based on the design-based replicate-weight confidence interval. PM = ab/c and is interpreted descriptively rather than causally.

**Table 8:** Hypothesis testing summary.

Hypothesis	Statement	Decision
H1	Family pressure is positively associated with psychosomatic symptoms.	Supported
H2	Family pressure is positively associated with mathematics anxiety.	Supported
H3	Mathematics anxiety is positively associated with psychosomatic symptoms.	Supported
H4	Mathematics anxiety is statistically associated with an indirect pathway between family pressure and psychosomatic symptoms.	Supported (design-based 95% CI excludes zero)

Hypothesis decisions are based on PISA design-based estimates using final and replicate weights. The cross-sectional indirect association is not interpreted as proof of a causal mechanism.

#### 4.2.1 Path a: family pressure → mathematics anxiety

The mediator model explained approximately 7.2% of the variance in mathematics anxiety in the equal-economy summary (*R*^2^ = 0.072, SE = 0.008). Family pressure was positively associated with mathematics anxiety (B = 0.187, SE = 0.021, *t* = 9.103, *p* < 0.001). The corresponding economy-specific estimates were B = 0.152 (SE = 0.030) in Hong Kong and B = 0.222 (SE = 0.028) in Macao. Thus, the positive path-a association was present in both economies.

#### 4.2.2 Path b and the direct association (c′): family pressure and mathematics anxiety → psychosomatic symptoms

The psychosomatic-symptom model explained approximately 10.2% of the variance in the equal-economy summary (R^2^ = 0.102, SE = 0.010). Mathematics anxiety was positively associated with psychosomatic symptoms after adjustment for family pressure and covariates (B = 0.159, SE = 0.014, t = 11.105, *p* < 0.001). Family pressure also retained a positive direct association with psychosomatic symptoms (c′: B = 0.122, SE = 0.016, t = 7.693, *p* < 0.001). In Hong Kong, b = 0.147 (SE = 0.020) and c′ = 0.142 (SE = 0.019); in Macao, b = 0.172 (SE = 0.020) and c′ = 0.103 (SE = 0.025). The direction of both focal paths was consistent across economies.

Gender remained associated with both outcomes: with coding 1 = female and 2 = male, negative coefficients indicate lower predicted mathematics-anxiety and psychosomatic-symptom scores for males. In the equal-economy summary, the male coefficient was −0.475 (SE = 0.034) for mathematics anxiety and −0.352 (SE = 0.029) for psychosomatic symptoms. Immigrant background was represented with separate second-generation and first-generation indicators using native students as the reference category.

#### 4.2.3 Total association model

The design-adjusted total association between family pressure and psychosomatic symptoms was B = 0.153 (SE = 0.017, *p* < 0.001). This estimate is consistent with the decomposition of the total association into direct and indirect components.

For practical effect-size interpretation, fully standardized focal coefficients were calculated from the economy-specific W_FSTUWT-weighted standard deviations and the design-based unstandardized coefficients. In Hong Kong, beta_a = 0.109, beta_b = 0.164, beta_c’ = 0.114, and beta_c = 0.132; in Macao, beta_a = 0.168, beta_b = 0.197, beta_c’ = 0.090, and beta_c = 0.123. The corresponding equal-economy averages were beta_a = 0.139, beta_b = 0.181, beta_c’ = 0.102, and beta_c = 0.127. The fully standardized indirect association averaged 0.026 across the two economies. These values confirm that the focal associations are small to modest in magnitude despite being statistically distinguishable from zero.

### 4.3 Direct, total, and indirect effects

The design-based equal-economy indirect association was ab = 0.0303 (SE = 0.0044), with a 95% BRR-Fay confidence interval of [0.0217, 0.0388]. The value 0.0303 is the equal-economy average of the economy-specific product terms (Hong Kong, ab = 0.0224; Macao, ab = 0.0382); it is therefore not expected to equal 0.187 × 0.159 = 0.0297, which is the product of the separately averaged path coefficients. Because the design-based interval excludes zero, the indirect association through mathematics anxiety is statistically distinguishable from zero. The total association was approximately 0.153 and the direct association was 0.122.

The corresponding proportion mediated was approximately 19.8%. This percentage is presented only as a descriptive decomposition of the cross-sectional association. Economy-specific indirect associations were also positive: Hong Kong, ab = 0.0224, 95% CI [0.0105, 0.0342]; Macao, ab = 0.0382, 95% CI [0.0259, 0.0504]. Thus, the result was not driven by one economy alone. In a mathematics-achievement sensitivity analysis using all ten plausible values, the indirect association remained evident (ab = 0.0275, SE = 0.0046, 95% CI [0.0185, 0.0365]) (Figure 1).

**Figure 1:**
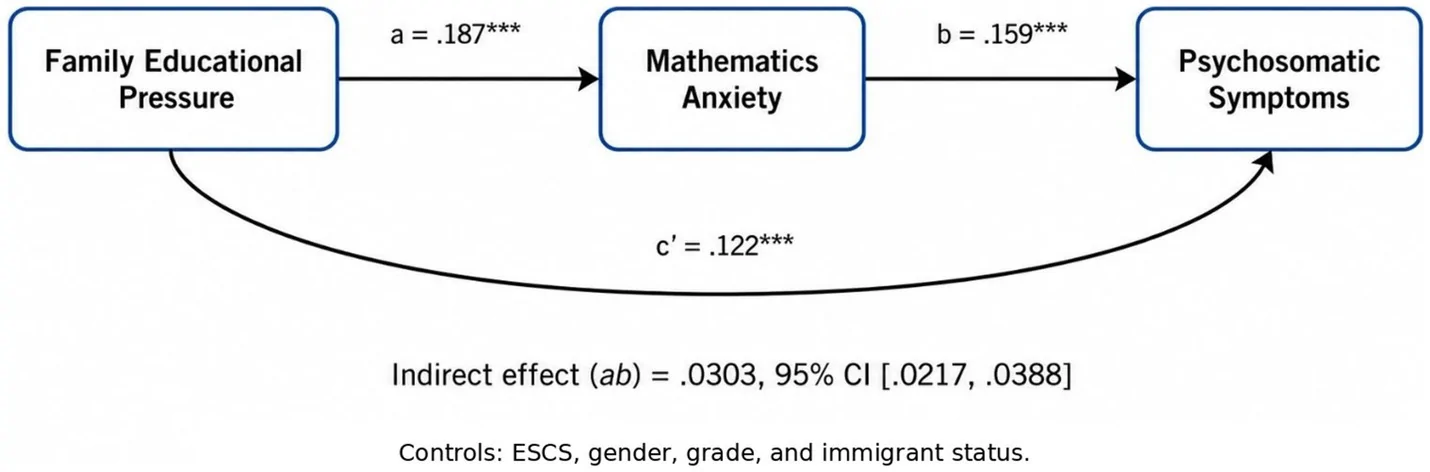
Statistical indirect association between family pressure, mathematics anxiety, and psychosomatic symptoms. Values are unstandardized design-based coefficients. Covariates (ESCS, gender, grade, first-generation immigrant status, and second-generation immigrant status) are omitted from the diagram for clarity. The indirect-effect CI is based on the 80 BRR-Fay replicate weights. ***p < 0.001 for paths a, b, and c′.

### 4.4 Hypothesis testing summary

All four hypotheses were supported as associational hypotheses. H1 was supported by the positive total association between family pressure and psychosomatic symptoms (c = 0.153, *p* < 0.001), with the direct association remaining positive after adjustment for mathematics anxiety (c′ = 0.122, *p* < 0.001). H2 was supported by path a (B = 0.187, *p* < 0.001). H3 was supported by path b (B = 0.159, *p* < 0.001). H4 was supported by the design-based indirect association (ab = 0.0303, 95% CI [0.0217, 0.0388]).

## 5 Discussion

This study examined whether perceived family educational pressure is associated with psychosomatic symptoms in the final analytic sample and whether mathematics anxiety is statistically associated with an indirect pathway between them. After re-estimating the models with the PISA final student weight and all 80 BRR-Fay replicate weights, all four hypothesised associations remained statistically supported. The design-based indirect association was 0.0303 (95% CI [0.0217, 0.0388]), and the pattern was positive in both economies. These results are modest in magnitude and are interpreted as cross-sectional associations rather than causal effects.

### 5.1 Theoretical interpretation

The findings are generally consistent with cognitive appraisal theory (Lazarus and Folkman, 1984), but the theory should be interpreted as an explanatory lens rather than as a mechanism established by these data. Family pressure, operationalized as agreement with a PISA item about feeling pressure from family to follow a specific post-compulsory-education path, was positively associated with mathematics anxiety. Because appraisal and coping resources were not measured, this association cannot show that students necessarily evaluated family demands as threats. Likewise, the positive association between mathematics anxiety and psychosomatic symptoms is compatible with an appraisal-based account of distress but does not establish an appraisal-to-somatic causal process. The cross-sectional results therefore indicate a pattern consistent with the theory while leaving temporal ordering and third-variable explanations open.

The findings are also consistent with ecological perspectives that locate family communication within adolescents’ proximal developmental environment (Bronfenbrenner, 1979; Bronfenbrenner and Morris, 2006). The persistence of a direct association after mathematics anxiety is included indicates that the proposed indirect pathway does not account for the entire family-pressure association. This multi-pathway interpretation is consistent with evidence that parental educational anxiety can be associated with adolescent problem behavior through psychological control and parent–child relationship quality (Lu F. et al., 2025).

Within the expectancy-value framework (Eccles and Wigfield, 2002), family expectations may be relevant to the perceived importance and cost of academic outcomes. One plausible interpretation is that mathematics anxiety becomes more salient when mathematics outcomes carry high subjective importance; however, task value and perceived cost were not measured directly. Cross-national work in comparable Confucian-heritage systems provides contextual support for examining this possibility (Morony et al., 2013), but the present data do not test a culturally specific mechanism.

### 5.2 Consistency with empirical literature

The results also confirm contemporary research findings based on the Chinese sample, but the differences in constructs should not be overlooked. In their study of 16,344 CEPS adolescents, Xu et al. (2024) demonstrated that higher parental educational expectations correlated negatively with mental problems, while higher academic pressure had positive correlations with mental problems. Academic pressure also moderated the relationship between expectations and mental health. It once again confirms the necessity to distinguish between expectations and pressure. Wang et al. (2025) confirm this finding with their longitudinal study, where they show that academic burden correlates with emotional problems in adolescents, while sleep disturbance, loneliness, and physical inactivity serve as statistically significant mediators. They support the idea that adolescent distress has several determinants, not just mathematics anxiety.

The results are also consistent with the current policy concerns about adolescent mental health issues. According to World Health Organization (2025a, 2025b), anxiety and depression are among the leading causes of illness and disability in adolescence and highlight the need for supportive environments at family, school, and community levels. The present results contribute to the existing literature by identifying one educational determinant of psychological health – family pressure perceived by students.

### 5.3 Boundary conditions and unexpected findings

Two covariate patterns are noteworthy but should not be overinterpreted. In the equal-economy mediator model, grade was negatively associated with mathematics anxiety (B = −0.067, SE = 0.026), whereas in the psychosomatic-symptom model it was positively associated with psychosomatic symptoms (B = 0.087, SE = 0.026). These coefficients describe adjusted cross-sectional grade differences and do not establish developmental change. Longitudinal research is required to determine whether mathematics-specific anxiety and broader somatic distress follow different trajectories across secondary-school grades.

Immigrant background was analyzed with native students as the reference category. First-generation status was negatively associated with mathematics anxiety in the mediator model (B = −0.320, SE = 0.054), whereas the second-generation coefficient was not statistically distinguishable from zero (B = −0.053, SE = 0.035). In the psychosomatic-symptom model, neither first-generation (B = −0.044, SE = 0.046) nor second-generation status (B = −0.013, SE = 0.032) was statistically distinguishable from zero. These are covariate associations; the study did not test immigrant status as a moderator of the focal pathways.

Accordingly, the study does not claim that the mediation pathway is invariant across gender or immigrant-background subgroups. Gender and immigrant background are adjustment covariates, while economy-specific estimates are reported to show whether the direction of the focal associations is consistent across the two analytic economies.

### 5.4 Magnitude of the indirect association and robustness

Mathematics anxiety accounted for approximately 19.8% of the estimated total association between family pressure and psychosomatic symptoms. Accordingly, most of the association was not accounted for by the proposed indirect pathway. Other processes—such as general test anxiety, perfectionism, fear of failure, academic burnout, sleep disruption, autonomy support, and school belonging—may also be relevant. For example, recent work links parent–child discrepancies in educational expectations with academic burnout (Fan and Hui, 2025). The 19.8% figure is a descriptive decomposition and should not be interpreted as a causal proportion transmitted through mathematics anxiety.

The mathematics-achievement robustness analysis using all ten PISA mathematics plausible values produced a similar pattern: a = 0.1504 (SE = 0.0211), b = 0.1800 (SE = 0.0144), c′ = 0.1298 (SE = 0.0167), c = 0.1573 (SE = 0.0162), and indirect association = 0.0275 (SE = 0.0046, 95% CI [0.0185, 0.0365]). The indirect association therefore remained evident after achievement adjustment.

### 5.5 Implications

Theoretically, the results are in line with the appraisal framework, under which the perception of family demands and mathematics anxiety are associated with poor psychosomatic well-being. Nevertheless, appraisal, perceived coping, task value, and cost have not been operationalized and thus not measured. Hence, the findings cannot prove any psychological mechanism but can be explained through an association pattern, which fits the framework of cognitive appraisal and beyond into the ecological and expectancy-value frameworks.

Practically, the results provide possible directions for further research, but do not prove that a particular intervention could help to change the situation. Schools might take into account the monitoring of mathematics anxiety and somatic well-being next to academic factors, while family communication and anxiety reduction in the classroom could be possible intervention sites. Their efficacy in this context has to be tested through longitudinal or intervention studies.

For families, the results reinforce the conceptual distinction between aspirational support and student-perceived pressure. Evidence from Xu et al. (2024) suggests that parental expectations and academic pressure are not interchangeable constructs. Family-facing initiatives may therefore focus on how educational goals are communicated, but any claim that a particular communication strategy reduces psychosomatic symptoms requires intervention evidence.

At policy level, the findings are consistent with a broader view of educational quality that considers student well-being alongside attainment (OECD, 2023b; World Health Organization, 2025a, 2025b). In two high-performing PISA systems, perceived family educational pressure showed a small positive association with psychosomatic symptoms, partly accompanied by mathematics anxiety. These findings motivate further longitudinal and intervention research rather than implying that system-level achievement itself causes psychological harm.

### 5.6 Limitations

Several limitations remain despite the design-based reanalysis. First, the data are cross-sectional, so temporal ordering and causal mediation cannot be established. Second, family pressure is measured with a single student-report item, which limits construct breadth and does not permit internal-consistency estimation. Third, the common mediation sample is restricted to Hong Kong and Macao because psychosomatic-symptom data were unavailable for Chinese Taipei. Fourth, although the newly conducted included-versus-excluded comparison showed only small observed differences in ESCS, gender, and relative grade (overall absolute SMDs = 0.025–0.054; economy-specific absolute SMDs ≤ 0.062), complete-case analysis can still be affected by differences on unobserved characteristics and no alternative imputation model was estimated. Fifth, although sample-specific internal-consistency coefficients are now reported for ANXMAT and PSYCHSYM, no study-specific measurement-invariance test was conducted across Hong Kong and Macao. Finally, the IDB “Table Average” is an equal-economy summary rather than a population-pooled coefficient, so economy-specific estimates should remain visible when interpreting the results.

Reverse and bidirectional associations remain plausible. For example, adolescents experiencing greater anxiety or psychosomatic distress may perceive family concern more negatively, or family members may respond to existing student difficulties with greater educational concern. Longitudinal data with repeated measurement are required to establish temporal precedence.

Another limitation concerns heterogeneous measurement quality across the focal constructs. Family pressure was measured with a single PISA student-report item (ST324Q05JA): “I feel pressure from my family to follow a specific path (e.g., go to college, work in the family business, learn a trade) after [the final year of compulsory education].” As a single item, FPRESS does not permit internal-consistency estimation and captures a specific perceived pressure concerning the student’s post-compulsory pathway rather than the full breadth of family academic pressure. By contrast, ANXMAT and PSYCHSYM are multi-item OECD-calibrated indices and show good sample-specific internal consistency in the present analytic file. The resulting unstandardized path coefficients therefore should not be compared as if all three constructs had identical scale precision or measurement error. Future studies should use broader multi-item measures for family pressure and formally test cross-economy measurement invariance.

The complete-case restriction reflects availability of the variables required by the model rather than any researcher-defined sampling fraction. The attrition diagnostic based on the pre-complete-case file found closely similar design-weighted ESCS, gender, and relative-grade distributions for included and excluded Hong Kong/Macao records, with all overall absolute standardized mean differences below 0.06. This evidence reduces concern about substantial observed selection on these measured covariates, but it does not prove a missing-completely-at-random mechanism or exclude selection on unmeasured factors. Future work could therefore evaluate robustness using multiple imputation or other missing-data strategies where methodologically appropriate.

The indirect association accounted for approximately 19.8% of the total estimated association, leaving most of the association outside the mathematics-anxiety pathway. Future studies should examine additional mediators and moderators and should test whether the observed economy differences replicate in independent samples.

## 6 Conclusion

A statistically distinguishable cross-sectional indirect association between perceived family educational pressure and psychosomatic symptoms through mathematics anxiety was observed among PISA 2022 students in Hong Kong and Macao. Using the PISA final student weight and 80 BRR-Fay replicate weights, family pressure was positively associated with mathematics anxiety (a = 0.187, SE = 0.021), mathematics anxiety was positively associated with psychosomatic symptoms (b = 0.159, SE = 0.014), and the direct association remained positive (c′ = 0.122, SE = 0.016). The equal-economy indirect association was 0.0303 (SE = 0.0044, 95% CI [0.0217, 0.0388]), corresponding descriptively to about 19.8% of the total estimated association. Economy-specific indirect associations were positive in both economies, and the robustness analysis using all ten mathematics plausible values yielded a similar indirect-association estimate.

These findings indicate that system-level academic achievement should not be conflated with individual psychological well-being. The observed associations are small to modest and cross-sectional; the standardized focal coefficients are consistent with that interpretation. Perceived family educational pressure and mathematics anxiety were positively associated with psychosomatic symptoms in the two economies examined, but the design does not establish predictive or causal precedence. Further longitudinal, multilevel, and intervention studies are required to test temporal mechanisms, competing mediators, measurement equivalence, and generalizability beyond Hong Kong and Macao.

## Data Availability

Publicly available datasets were analyzed in this study. This data can be found here: data is available on reasonable request from the authors.

## Appendix A: PISA 2022 measurement item wording

The item wording and response categories below are reproduced for measurement transparency from the PISA 2022 questionnaire and technical documentation (OECD, 2023a; OECD, 2024a, 2024b).

**Table A1:** Mathematics anxiety (ANXMAT; ST292).

Item code	Wording
ST292Q01JA	I often worry that it will be difficult for me in mathematics classes.
ST292Q02JA	I get very tense when I have to do mathematics homework.
ST292Q03JA	I get very nervous doing mathematics problems.
ST292Q04JA	I feel helpless when doing a mathematics problem.
ST292Q05JA	I worry that I will get poor [marks] in mathematics.
ST292Q06JA	I feel anxious about failing in mathematics.

Response categories in the public-use file are coded 1 = Strongly agree, 2 = Agree, 3 = Disagree, and 4 = Strongly disagree. The released ANXMAT WLE index is oriented so that higher scale scores indicate greater mathematics anxiety.

**Table A2:** Psychosomatic symptoms (PSYCHSYM; WB154).

Item code	Item wording	Question stem
WB154Q01HA	Headache	In the past six months, how often have you had the following?
WB154Q02HA	Stomach pain	
WB154Q03HA	Back pain	
WB154Q04HA	Feeling depressed	
WB154Q05HA	Irritability or bad temper	
WB154Q06HA	Feeling nervous	
WB154Q07HA	Difficulties in getting to sleep	
WB154Q08HA	Feeling dizzy	
WB154Q09HA	Feeling anxious	

Response categories: Rarely or never; About every month; About every week; More than once a week; About every day. Higher PSYCHSYM scores indicate more frequent psychosomatic symptoms.
